# The Section on Stomatology as a Factor in the Evolution of Dental and Medical Science*Read before the Section on Stomatology at the Sixty-Fifth Annual Session of the American Medical Association, Atlantic City, N. J., June, 1914

**Published:** 1915-01

**Authors:** George V. I. Brown


### THE SECTION ON STOMATOLOGY AS A FACTOR IN THE EVOLUTION OF DENTAL AND MEDICAL SCIENCE.*

BY GEORGE V. I. BROWN, M.D.

Recent advancement toward more general recognition of the pathologic influence of oral affections as factors in disease has been so great that almost every thought of distinction between those who treat disorders of the teeth, and the practitioners whose ministrations are directed to the relief of such distress in other anatomic regions, or of the organism as a whole, has been swept as driftwood before a tidal wave in the common desire of a great humanitarian impulse that brooks no trammel of limitation by degrees or titles.

Surely this is an age when dreams come true, for the visions that inspired the speech and directed the pens of such men as Alkinson of New York, Garretson, Bonnwill and Flagg of Philadelphia, Allport of Chicago, and their contemporaries in early years touching as they then did the very boundary limits of the hights of empiricism, are now becoming prophetic in the light of actual scientific demonstrations of their truth.

In like manner the foundation laid with the help and guidance of the master surgeon, Samuel D. Gross, and the great builder of medical associations, Dr. N. S. Davis, and the little band of dentists who joined hands as organizers

* Read before the Section on Stomatology at the Sixty-Fifth Annual Session of the American Medical Association, Atlantic City, N. J., June, 1914.

of the Section on Dentistry, now the Section on Stomatology of this great body, the American Medical Association, has become the underlying support of a rapidly extending scientific edifice. It is obvious that this superstructure can be sustained only by an ever-increasing broadness at its base; otherwise this influence must perish. It therefore seems advisable at this time to glance backward along the recorded pages of the thirty-two years of the Section's existence, and then to scan as best we may the already forming, though ill-defined, outlines of the possible future requirements in this division of medical science.

At a meeting of the American Medical Association held at Richmond, Va., May 3, 4, 5 and 6, 1881, there gathered together for the purpose of forming a Section on Dental and Oral Surgery, the following organizers: Drs. W. W. Allport, Chicago; Huxhurst, Grand Rapids, Mich.; Eugene S. Talbot, Chicago; T. W. Brophy, Chicago; J. A. Williams, Boston; George L. Parmlee, Hartford, Conn., and D. H. Goodwille, New York. At that meeting Dr. Samuel D. Gross of Philadelphia asked for a suspension of the regular order of business and moved that the By-Laws of the Association be so amended as to create another Section to be known as No. 7, and entitled Dentistry. This was made effective the following year.

The first meeting was held at St. Paul, June 6, 1882. It is especially interesting in view of later events to note that at this time Dr. Allport offered a resolution providing for the appointment of dentists in the Army and Navy and to co-operate with Dr. Maynard of Washington for this purpose.

The resolutions of sympathy spread on the records of the Section, as they from time to time mark the passing of some important member, bear the stamp of a depth of feeling that can not be measured by ordinary standards because each one of these far-seeing men was an integral part of the little group that was laboring for higher things in the progress of dental science.

My purpose in presenting this brief reference to the past is not so much an endeavor to do justice to faithful individual effort during a period of thirty-two years, deserving of such praise as the work may be, as to try to present in just the right way, if possible, the whole scheme of the present status of the pathology and treatment of tooth and mouth affections in their common relation to other diseases and incidentally to assist in clearing up the confusing conditions which pertain to the professional association of those who treat them, whether distinguished by the degree of D.D.S. or M.D., or both.

I fully realize that it will be next to impossible for me to state in exact terms just what this relationship, or state of interdependence, should be, for no one really knows and no two of those who ought to be best informed have ever been able to state their views similarly. Yet for this very reason there is even greater need of proper treatment of this subject.

To picture comprehensively the real influence of the work of this Section, one should have in mind, first, the all-important part that the mouth plays in the etiology of disease, as a source of focal infection, a cause of local or general nerve disturbances, and a factor in developmental, digestive, circulatory and other disorders; second, a just idea of the constructive benefit of the steadily continuous presentation of these subjects during thirty-two years in a section of the largest and best-recognized medical organization in this country, and of calling the attention of medical practitioners to this particular field in a specific way through the medium of their own medical journal; third, it should be understood that in the past too much attention has been given to degrees and much harm done through failure of dentists generally to realize that strictly dental organizations bear precisely the same relation to the Section on Stomatology of the American Medical Association that the special societies of other divisions of practice bear to their respective associations.

For example, the sections on surgery, internal medicine, eye, ear, nose, and throat have their corresponding associations in the American Surgical Association, American Neurological Association, American Laryngological, Rhinological and Otological Society, American Dermatological Association, American Association of Obstetricians and Gynocologists, etc., just as the dentists have the National Dental Association. There should be no question of loyalty involved in consideration of attendance on both.

When the members of the National Association of Dental College Faculties voted to extend the courses in their colleges to four years, the American Medical Association promptly accepted dentists to full membership in this body; but when the colleges decided to go back to three-year courses for graduation, it was obvious that there must be some distinction between men who have had four years' training when considering their association with those who have had only three. Thus dental graduates came to be accepted as associate members, but there is no appreciable difference between these two forms of membership in the conduct of section work and there ought to be a more general understanding of the fact that every dentist in this country has profited in some degree at least, professionally, socially and even personally, by the good work that this Section has accomplished.

There are grave questions to be met if dentistry is to hold her own in the advancement which marks the modern appreciation of pathologic and therapeutic considerations. This is no time to cavil about degrees. The true standard in rating the future practitioner in that branch of the healing art which directly relates to oral affections must be the measure of his knowledge of medical science, and his ability to cope with the requirements of a more or less radically changed order of practice.

From an educational point of view this means that the formerly so-called theoretical branches of the curriculum of his special college courses are after all in reality the very practical ones. Students of dentistry can no longer be permitted to skim through these studies and pass out into active practice with only a superficial knowledge of them. Anatomy, physiology, physics, chemistry, biology, histology and pathology are quite as necessary for them as for their brother students in general medicine. Obviously this can not be accomplished under the plan of instruction as now given in preparation for the D.D.S. degree.

If, as Rosenow and other investigators have apparently proved, the principal sources of focal infection are found in the tonsils, the mouth, teeth, nose and nasal accessory sinuses, and if, as he also states, pathogenic micro-organisms can be harbored in these situations during the period of transmutation by which they may assume an increased virulence which makes them accountable for such diseases as ulcer of the stomach, rheumatism and its well-known chain of far-reaching symptoms, for endocarditis, myocarditis, gall-stones, and joint affections, and if in addition it can be shown, as we believe it can, that by proper care of the teeth and direction of the development of the dental arches, the pathologic conditions of both the tonsils and the nasal accessory sinuses can in large measure be controlled or prevented, then it must be accepted that full medical training is as essential for the one who has charge of the welfare of this fertile field for the propagation of disease, as it is for those who undertake to treat the general body manifestations thereof.

This does not of necessity mean that a student must first pursue the regular four years' course in medicine with the additional premedical and postgraduate years of studenship that are now being urged as necessary for proper training for the practice of medicine, and in addition a full course of two or three years more to complete his equipment for the practice of dentistry.

Desirable as these long years of preparation may be, and admitting to the fullest extent the value of the knowledge that might thus be acquired, there are nevertheless certain definite reasons why such preparation for the practice of dentistry is not practicable under present educational conditions.

Lest my position be misunderstood in view of this statement, and others that follow, it seems advisable for me to state that according to my belief it will no more be possible for dentistry to continue permanently as a profession distinctly apart from medicine than it was for the United States to exist half slave and half free, as recognized by Lincoln.

There must ever be some common meeting ground for all who practice legitimately every branch of the healing art. That has been, and will be with steadily growing recognition of its importance, the office of this, the representative stomatologic division of this great medical body.

Beyond a doubt there must come in the course of time the adoption and general use of degrees to distinguish the several medical divisions. Men without special training in surgery can not be permitted to continue indefinitely to undertake grave surgical operations simply because they have qualified to the extent of a medical degree and a state board examination of general character. This is equally true of eye and ear, or nose and throat, or other limited practitioners, and they also should be required to have distinct evidence of preparation for their particular field of work.

With the continued extension of the required number of years of medical study and the very evident need of additional training for special practice, it would seem that in the course of the evolution of educational conditions there would ultimately be established a fundamental course of study which all must complete before taking up a special division of practice. The number of years required for this foundational preparation would be somewhat less instead of more than is now required for the M.D. degree, and the student after having completed this course could then in two years more of special study prepare himself for the degree which would represent his actual life work more completely than is usual at the present time. The following opinion expressed by Dr. E. C. Kirk is of interest because he is in charge of the Thomas W. Evans Museum and Dental Institute and therefore in control of the largest structure in the world exclusively devoted to dental instruction. As this institute is designed to give both undergraduate and postgraduate instruction, the views of one who is to direct its course are important in this relation.

"It seems to me to be inevitable that in the course of time a rearrangement of the medicinal curriculum must result from the specializing tendency, and from the increase of bulk in medical knowledge, both of which factors have made it impossible to educate any man in medicine so that he can cover the whole ground.

"That reorganization will, I think, take something of the following form: A sufficient length of time in the medical curriculum will be devoted to the training of men in all of the sciences that are fundamental to the entire field of the science and art of healing, and the termination of that phase of the curriculum will be marked by the conferring of a degree which will be educationally equivalent to the bachelor's degree in science and letters, and from the point of attainment of the bachelor's degree in medicine the student will specialize in groups of studies that will make him an efficient practitioner in some recognized special department of medicine at the termination of which he will have conferred on him his doctorate in medicine, and he will be licensed to practice only within the limits of the specialty for which he has been trained. Should he desire to acquire the right to practice an additional specialty it will be necessary for him to prepare himself by further educational work in another special department, and then secure the license to permit him to practice in that department, and so on."

It may be too much to demand such sweeping reforms in medical education until such time as in the course of natural evolutional progress these changes gradually may be inaugurated. There is, however, an immediate necessity for more thorough education of students who are to become dental practitioners in the branches of study in which their courses parallel those of their brothers in medicine. Without delay these fundamental medical sciences should be given exactly the same as for students of general medicine, and a passing mark on any one of them should be considered equivalent in every way, no matter what branch of practice may be contemplated or selected. Thus, the dental graduate will not only be more thoroughly prepared for his special division of practice, but he will also have so many definite credits toward the medical degree that he may reasonably be able to complete this course also without too much hardship. In due time it will follow that increasing numbers of the most promising students will complete their double education and take both degrees. Ultimately through the selective influence of a more enlightened laity the two degrees will be required.

Dealing practically with the educational difficulties that lie in the way of idealization in this educational formula, we find that in the absence of certain necessary but apparently impossible changes in the underlying primary courses of instruction, by the time the average student could complete his literary course with four years leading to a degree in arts, or science, and then four years in medicine, with the very necessary hospital internship of one year more, in a majority of cases he will have reached such an age that the first enthusiasm of his career will be lost. It is a grave question if the average result would be as good as it has been in the past when an aspirant for a medical degree was allowed to enter the medical college with but little if any more preparation than a high-school education. But leaving that much-discussed question for the settlement of educators now battling with it, there at least can be no question regarding the probability that at this advanced period of life it would as a general rule be quite impossible for an individual to acquire the necessary manual dexterity for successful dental practice. It would seem then that the best that can be done at the present time is so to arrange the curriculum of the dental student as to make it possible for him to have full training in the technic laboratories from the very beginning of his course, and to take during his first two years as many of the strictly medical studies as he can carry and complete in each the full regular medical student's course in these branches.

Obviously when the medical student's first and second years are crowded to the limit of average capacity, the dentist can not do all this thoroughly and spend approximately forty hours a week in dental technic laboratories beside. Every teacher of anatomy or physiology or other similar branch can testify to the difficulties that have presented themselves when this has been attempted. By distributing the work and making the courses for the dental degree extend through four years instead of three, then with all this work out of the way, one year more could be made sufficient for acquirement of the medical degree.

Our problem is thus a problem no longer and the dentist is now a completely developed specialist in his work. Whatever the final solution of the complex professional relationship may be, it is at least apparent that the influence of this section, past, present and future, will be instrumental in its final adjustment.

The horizon from a dental and oral outlook appears wonderfully bright at the present time. The establishment of free dental clinics in many of the principal cities of the United States, examinations of the mouths of school-children, lectures on oral hygiene under state appointment; endowed institutions for the care of the mouths and teeth of the poor and the systematic education and training of dental original research workers, supported by funds contributed for the purpose, are some of the many evidences that might be cited of the undisputed right of this stone rejected by the medical builders in 1840 to a place in the great temple of medical science.

Who can doubt that the cumulative effect of the long years of faithful educational service that the members of this Section have rendered has been a potent influence in the preparation of the soil in which the seeds of the various movements have given such gratifying evidence of fertility? Above and beyond all other factors, the usefulness of the work of this Section must be attributed to our secretary, Dr. Eugene S. Talbot, who has made it his life work, and who for years has directed the broadness of the scientific policy that has shaped the character of the program from year to year.

Next to this the most active influence has been the earnest co-operation and assistance of Dr. Simmons and his predecessor, Dr. Hamilton, as editors of *The Journal* of the American Medical Association, without which there could have been no such wide spreading of the truths of the gospel of oral medicine.

For the future the most desirable consideration appears to be a closer union between this Section and the state, county and national dental organizations such as may lead to increased co-operative activity. This would also lead to a more widely extended recognition of the value of this link in the chain of medical specialties which has been so carefully welded and forged and tried in the fire of these many years, in order that it might be relied on to hold with sufficient security its own representative division in proper relation to all other parts of the associated body of the sacred order of those whose mission is to mitigate and relieve the ills of suffering humanity, whose common purpose is to render human life happier if not longer and at least a little more worth the living.

445 Milwaukee Street.

ABSTRACT OF DISCUSSION.

Dr. Joseph Head, Philadelphia: Dr. Brown's paper states clearly a fundamental thought that has been in the mind of every intelligent dentist since he began to practice. The dentist ought to be so perfect a mechanic that such perfection would be considered a goal easily sufficient for a life occupation; but the dentist also should be a thoroughly trained physician. We find that the mechanical appliances of dentistry which heretofore have been considered independent of a knowledge of medicine are now so closely associated with the health and happiness of the community that they can no longer be considered apart from pathology and therapeutics, but all these must be considered as an independent whole. For just as general medicine contains specialties, a complete comprehension of which would require twenty-five or thirty years to master, so the dentist has undertaken a specialty the complete comprehension of which would require ten or twelve years. This preliminary training is not feasible. By the time a man has studied ten or twelve years in college he will be so old that the enthusiasm of youth will be apt to be lacking and so it would be difficult for him to build up a practice. There should be, in my opinion, a bachelor's degree of medicine that *per se* would not allow any man to practice medicine, but from which he could go on to the special doctor's degree to which he would effectively confine his efforts.

Dr. F. B. Moorehead, Chicago: The discussion of any educational subject is of vital human interest and no set of men can ever determine the ultimate policy of any educational system, simply because it is a matter of evolution. This whole question that we are facing to-day in the matter of dental and medical education is simply a sign of the times. As man expands in thought and usefulness in the world, he is most surely going to demand a different type of training, and that is what this means. The question of dental education is a live subject to-day, because we are now facing a fact and not a theory. The physician and dentist have both awakened to the fact that the mouth is more important than it was thought in the past, and, therefore, the dentist must face and solve some serious problems. This calls for a different type of dental student.

How to make that different type is an exceedingly difficult problem. We must educate enough dentists to care for the people and at the same time we must change from the kind of education we have given in the past and make the dental student a broader man. One step in that direction is the four-year course which I am convinced is sure to come. The first year in the four-year course should be a predental year in the university. The university method of study; the university enthusiasm and spirit will give the student a bigger and better perspective. This is the type we must have for the dentist of to-morrow. One can not get away from the fact that, as much as we value medical training, the man who is to be a good dentist must be a good mechanic, because if he is not he can not make his patient comfortable, and unless he makes his patients comfortable he is not serving them properly. The man who is uncomfortable is physically and mentally unfit. So we can not lose sight of the fact that we must make mechanical dentists while developing the man of parts to take care of the medical side of dental practice.

Dr. Arthur D. Black, Chicago: I think Dr. Brown has presented the consensus of opinion of a great many physicians and dentists as to the general proposition of the future of our educational system in medicine and dentistry, and the relationship which will probably exist between dentistry and the other specialties on the one hand, and a general medical education on the other. By the process of evolution we are gradually coming to the point where, whether it is taught in the medical school or in the dental school, all men who practice medicine and the specialties of medicine must have the same foundation education. It is certainly as important that the dentist know as much of general medicine as the man who is practicing any other specialty of medicine. That it will be a great many years before the dental specialist gets that foundation in the medical school, I am quite thoroughly satisfied. The greatest problem that we have from the point of view of dental education is that of developing a different type of men in the dental school, men who will enter with the idea that they will have to study and master physiologic and pathologic problems as a basis for their technical service. In the school with which I am connected we are endeavoring to develop this type under the conditions which now necessarily exist by shifting more of those subjects which require hard study into our freshman year and diminishing the amount of technical work required during that year. We hope to impress the student with the fact that he must learn to study if he ever gets the dental degree, and at the same time we are eliminating in the freshman year a lot of men who will not study. Doubtless the higher entrance requirement, especially if this consists of a prescribed course of a year in college, will be of material aid, both in the extra year's work and the college associations. This is a problem which many men interested in college work are considering at present; that we should prescribe, previous to admission into the dental school, not only a high school education, but definite instruction in certain branches which the academic department is better qualified to give than is the dental school.

There is, however, another question to be considered in this connection. While the dental school must develop men who will be able to solve the more complex problems involved in the relationship of mouth conditions to general health, they can not, on the other hand overlook the greatly increasing demand for the ordinary care of the teeth. The wave of public education in oral hygiene has brought such an increased demand for the most ordinary dental service that we can not go so fast in this matter of advancing dental schools that we will materially reduce the number of graduates and thus not provide sufficient men for the ordinary dental service.

Dr. Eugene S. Talbot, Chicago: At the time this section was established few graduates of medicine were practicing dentistry. Indeed very few were graduates in dentistry, as compared with those practicing without a degree. The friendly relations between dentistry and medicine, and between graduates of dentistry and non-graduates did not then exist. The medical profession on the other hand has always shown the greatest respect for our specialty. This is no doubt due to the fact that physicians realize that each branch of medicine has gradually advanced from small beginnings, requiring many years to reach the present state of evolution. They are willing and glad to help the youngest branch of medicine in its rise to a higher position. The Section on Stomatology has from year to year endeavored to represent the best material in the dental profession. We had been organized only a few years when we found that some of the ablest men practicing dentistry had not the medical degree, which was then necessary for membership in the A. M. A. We petitioned the general body and they immediately changed the constitution and by-laws so that practitioners of dentistry, holding the degree of D.D.S. and belonging to their state and local dental societies, could become ''dental members'' enjoying all the privileges of regular medical members. Again the medical profession came to our rescue and gave us a helping hand. Dental members have been stimulated to a higher sense of their relation to medicine. On the other hand, at nearly every meeting the names of physicians and surgeons are on the program. At present physicians are invited to read papers in dental societies and dentists have read papers at medical gatherings. There is no doubt that, in the future, some medical societies will recognize the dental degree for all that it implies, just as they now recognize other specialties in medicine. Recently a mutual ground between dentistry and medicine has been found, for which this section stands and which neither dentistry nor medicine are in themselves qualified to practice or teach. Those who will be the future successful practitioners of stomatology must be well grounded in the general principles of medicine. The practitioner of medicine has already begun to recognize those specialists who are abreast of the times with regard to mouth infection as a source of systemic disease. The physician will soon seek those who are competent to assist him in making an exact diagnosis. Dentists who wish to be successful in the future must thoroughly educate themselves in both medicine and dentistry. The Council of Medical Education should discourage medical schools from conferring medical degrees on dentists who have not taken the prescribed medical course.

Dr. W. C. Fisher, New York: Dr. Brown's suggestion as to the establishment of the B.M. degree is a good one. I read a paper last year entitled, "The Need of a New Degree, Bachelor of Medicine." I simply gave it as a suggestion at that time, and backed up my statements by those of Dr. Bevan of the Council on Medical Education of this Association, in which he stated that too much time was being wasted in preliminary preparation before men entered the medical schools; that we could not turn out competent medical men in twenty-nine or thirty years with the present restrictions, and that some new scheme would have to be devised whereby men could go out on their life work earlier. Professor Lowell of Harvard has said that instead of demanding the Bachelor of Arts degree before entrance to medical schools it would be better if we would incorporate some of these studies in the course leading to the Bachelor of Science degree and take one year from the four-year course, instead of spending a couple of years in studies that did not particularly relate to medical education. The man who has an excellent preparatory school education, and that does not mean two years in college, is qualified to undertake the study of the sciences of medicine and dentistry. When we speak of getting better men I am in favor of that, but I am opposed to anything that makes it so late in life before a man can acquire that degree which entitles him to go forth into the world and earn his livelihood.

Dr. G. V. I. Brown, Milwaukee, Wis.: We have been discussing subjects that my paper relates to ever since the meeting opened, and it is clear to me that, in the settlement of this question, the time element alone will not do it, but it is equally clear to me that we should not make any step in this direction until we know best what to do. —*Journal A. M. A.* December 12, 1914.

